# Serum apelin is associated with left ventricular hypertrophy in untreated hypertension patients

**DOI:** 10.1186/s12967-015-0635-5

**Published:** 2015-09-05

**Authors:** Lijun Ye, Fenghua Ding, Liang Zhang, Anna Shen, Huaguo Yao, Liehua Deng, Yuanlin Ding

**Affiliations:** Department of Critical Care Medicine, Affiliated Hospital of Guangdong Medical College, No. 57 Southern Renmin Avenue, 524023 Zhanjiang, Guangdong China; The Institute of Medical System Biology, School of Public Health, Guangdong Medical College, Dongguan, China; Department of Cardiology, The Third Affiliated Hospital of Southern Medical University, No.183, West Zhongshan Ave, Guangzhou, Tianhe District China

**Keywords:** Apelin, Left ventricular hypertrophy, Hypertension

## Abstract

**Background:**

Apelin is an endogenous ligand for the G protein-coupled receptor APJ. The association between apelin and cardiac modeling has been reported. However, if serum apelin affect the left ventricular hypertrophy (LVH) prevalence in hypertensive patients remains unknown.

**Methods:**

We enrolled 344 untreated hypertensive patients. The presence of LVH was determined by echocardiography. The blood was drawn from these patients and serum apelin level was detected. To study the direct effect of apelin on cardiac hypertrophy, cardiomyocytes were cultured and were transfected with apelin gene. Morphometric analysis and measurement of protein contain per cell were then performed.

**Results:**

We observed a significantly lower serum apelin level in hypertensive patients with LVH compared with those without LVH. Receiver operating characteristic analyses shows that serum apelin level is robust in discriminating patients with LVH from those without. Our in vitro study showed that cellular protein content and cellular size was increased by Ang II treatment, which can be markedly inhibited by the apelin over-expression in cultured cardiomyocytes.

**Conclusion:**

Our clinical date established a link between apelin and LVH, suggesting serum apelin may be used as a predicator for LVH prevalence in hypertensive patients. The direct evidence in vitro suggest apelin pathway is involved in the cardiomyocyte adaption to hypertrophic stimuli.

## Background

Left ventricular hypertrophy (LVH) is one of the major complications of hypertension [1, 2]. The prevalence of LVH is associated with significantly higher rate of cardiovascular disease, including angina pectoris, myocardial infarction, heart failure, cerebrovascular accidents, and sudden death [3]. Currently there is no reliable method to predict the prevalence of LVH in hypertensive patients. It is important to discover new markers which can identify patients prone to develop LVH.

Apelin is an endogenous ligand for the G protein-coupled receptor APJ, which is synthesized as a 77-amino acid prepropeptide. Apelin/APJ system exerts a variety of cardiovascular effects. A recent study shows that the cardiac apelin is markedly down-regulated in experimental heart failure (HF) animal [4]. Apelin gene therapy increases myocardial vascular density and ameliorates diabetic cardiomyopathy via upregulation of sirtuin 3 [5]. Apelin protects the brain against ischemia/reperfusion injury through activating PI3K/Akt and ERK1/2 signaling pathways [6]. Apelin also plays an important role in atherosclerosis development in mice [7]. Myocardial injection of apelin-overexpressing bone marrow cells improves cardiac repair via upregulation of Sirt3 after myocardial infarction [8]. Apelin could reduce risk of contrast-induced nephropathy in patients with congestive heart failure [9].

To date, apelin has been regard as a potential marker of coronary artery stenosis and atherosclerotic plaque stability in ACS patients and heart failure patients [10]. However, the association between the serum apelin and LVH in hypertensive patients remains unknown. In this study, we enrolled patients with essential hypertension (EH) and found that serum apelin is closely associated with the prevalence of LVH in these patients, suggesting serum apelin may be used as a marker to predict the LVH prevalence.

## Methods

### Enrollment

A total of 344 patients newly diagnosed with EH were recruited in our hospital from April 2008 to September 2013. All were untreated patients. Patients with secondary arterial hypertension, hypertrophic cardiomyopathy, valvular heart diseases, pulmonary hypertension, coronary heart disease, liver/kidney insufficiency, metabolic disease, and immunological diseases were excluded from this study. According to the presence or absence of LVH, subjects were divided into LVH+ (patients with LVH) and LVH− (patients without LVH) groups. A complete medical history was obtained from all subjects, including diabetes mellitus (DM), alcohol intake, cigarette smoking, weight, height, body mass index (BMI), systolic blood pressure (SBP), and diastolic blood pressure (DBP). BMI was calculated as following formula: BMI = Weight (kg)/(Height (m) × Height (m)) [11]. Biochemical variables including serum creatinine, total cholesterol (TC), total triglyceride (TG), high-density lipoprotein cholesterol (HDL-C), low-density lipoprotein cholesterol (LDL-C) were acquired from patient’s medical chart. The study protocol was approved by the ethics committee of our hospital. All patients provided an informed written consent.

### Measurement of LVH

We measured left ventricular end-systolic dimension (LVESD), left ventricular end-diastolic dimension (LVEDD), end-diastolic interventricular septal (IVS) thickness, and left ventricle posterior wall thickness (PWT) using ultrasonic Doppler echocardiography (Acuson Sequoia 512, Siemens Medical Solutions, Mountain View, CA, USA). Three successive cardiac cycles were studied and left ventricle ejection fraction (LVEF, %) and fractional shortening (FS, %) were obtained based on the data. LV mass = 0.8 × 1.04 [(IVS + LVIDD + PWT)3 − LVIDD3] + 0.6. LVMI = LVM/height2.7. Left ventricular hypertrophy was defined as LVMI >49.2 g/m2.7 in men and LVMI >46.7 g/m2.7 in women.

### Serum apelin, BNP and hs-CRP level detection

The peripheral blood samples were collected from all participants after 12-h overnight fast. Apelin was measured by an ELISA kit (Apelin-12, Phoenix pharmaceuticals, Belmont, USA). The sensitivity was 0.05 ng/mL, and intra- and inter-assay variations were <5 and <14 %, respectively. Serum high-sensitivity C-reactive protein (hs-CRP) measurements were performed using a commercially available high-sensitivity assay (Roche Diagnostics).

### Rat neonatal cardiomyocytes culture and treatment

The whole hearts from neonate rats were isolated, minced and rinsed in hood by using by using the method invented and patented by Dr. Xuwei Hou (patent number: CN 101955884 A and CN 101955884 B). This method can protect cardiomyocyte from excessive enzyme damage when being digested from cardiac tissue. Briefly, the whole hearts from neonate Wistar rats (age less than 3 days) were isolated, minced and rinsed in hood. Five to six cycles of digestion using collagenase (95 U/ml) (Sigma Aldrich, Bornem, Belgium) and 0.6 U/ml of pancreatin (Sigma Aldrich, Bornem, Belgium) were performed. At the end of each cycle, the suspension was centrifuged and the supernatant collected, pooled, centrifuged and resuspended in the cardiac medium containing DMEM and M199 (volume ratio: 4:1) [12].

### Apelin gene transfection

The apelin complementary DNA was purchased from OriGene (Rockville, MD, USA). The coding region was amplified with the following primers: 5′-CGCGAATTCGGCATGAATCTGCGGCTCTG and 5′-GCGCTCGAGTCAGAAAGGCATGGGTCC. The amplification products were subcloned into the pcDNA 3.1. vector using *Eco*RI and *Xho*I restriction enzymes (Invitrogen, Carlsbad, CA, USA). Cultured cardiomyocytes were transfected with a control or an apelin-encoding pcDNA 3.1 vector using the FuGENE 6 transfection reagent (Roche Diagnostic, Mannheim, Germany) according to the manufacturer’s guidelines. Stable transfectants were selected by their resistance to geneticin (400 µg/ml; GIBCO, Paisley, UK). After transfection, the cells were then treated with Ang II (500 nM) for 48 h to induce cellular hypertrophy [13].

### Morphometric analysis

Forty-eight hours after Ang II (500 nM) treatment, cellular hypertrophy was evaluated by measuring cardiomyocyte cell surface using a digital image analysis system (Leica QwinV3, Leica Microsystems Ltd., Cambridge, UK). To examine changes in cell morphology and cytoskeleton, cells were fixed in 4 %paraformaldehyde, stained with FITC-conjugated Phalloidin (Sigma) for 30 min and mounted in Vectashield with 4′,6-diamidino-2-phenylindole (Vector Laboratories, Peterborough, UK). Cellular hypertrophy was evaluated by measuring cardiomyocytes surfaces using a digital image analysis system (Leica QwinV3, Leica Microsystems Ltd., Cambridge, UK). Five random fields (with approximately 10–15 cells per field) from every sample were averaged and expressed as μm^2^/cell. All experiments were repeated three times.

### Measurement of protein contain per cell

The cultured cardiomyocytes were trypsinized and counted using a cell counting chamber (Beckman Coulter, Fullerton, CA, USA) and then lysed with RIPA buffer (Santa Cruz Biotechnology, Santa Cruz, USA). A total of 5 × 106 cells were used. The cell lysates were prepared to determine protein content by BCA protein assay kit (BioVision, Inc., Milpitas, CA, USA). Then the protein synthesis of cells was determined by dividing the total amount of protein by the number of cells, namely, protein per cell [14].

### Western blotting analysis for cardiomyocyte hypertrophy markers

Collected cardiomyocytes were separated by trypsin and the protein concentration in the supernatant was determined with a BCA protein assay kit (Beyotime, Jiangsu, China). The isolated protein (25 μg) from cardiomyocytes was separated by 10 % SDS-PAGE and transferred onto polyvinylidene difluoride nylon membranes. The blots were probed with anti-rat Apelin peptide (1:1000 dilution, Santa Cruz Biotechnology, Santa Cruz, USA), anti-rat atrial natriuretic peptide (ANP), Osteopontin (OPN) and transforming growth factor beta (TGF-β1) (all 1:1000 dilution, Millipore, USA), anti-GAPDH antibody (1:1000 dilution; Santa Cruz Biotechnology, Santa Cruz, USA). Then with horseradish peroxidase-conjugated secondary antibodies (1:5000 dilution), and visualized using an ECL detection kit (Amersham Biosciences, Piscataway, NJ, USA). The optical densities of the bands were quantified by densitometric analysis performed with a quantitative imaging system (Bio-Rad, USA). All western blot experiments were repeated three times.

### Statistical analysis

Data on quantitative characteristics are expressed as mean ± SD or mean. Data on qualitative characteristics are expressed as percent values or absolute numbers, as indicated. Differences in demographic characteristics and Analyses were performed using the software SPSS 16.0 (SPSS Inc., Chicago, IL, USA). All P values were two-sided, and a P value <0.05 was considered significant variables and the χ^2^ test for all categorical variables. Receiver operating characteristic (ROC) analyses was performed to determine a discriminative ability of apelin in LVH prevalence in these patients. We also performed a multivariate logistic regression with LVH+/LVH− as the dependent variable and serum apelin as a covariate adjusted at least for age, gender, BMI, diabetes, smoking, SBP, and serum lipids (HDL- and LDL-cholesterol). The data of in vitro experiments were compared with ANOVA analyses. Analyses were performed using the software SPSS 16.0 (SPSS Inc., Chicago, IL, USA). All P values were two-sided, and a P value <0.05 was considered significant.

## Results

Among all hypertensive patients, 98 were classified as hypertensive with LVH and 246 as hypertensive without LVH, according to the presence of LVH. There were no statistically significant differences between the two groups in age, sex, diastolic BP, TC, TG, LDL-C, and HDL-C (P > 0.05; Table 1). We found that LVH+ patients had higher percentage of smokers, higher BMI, systolic BP, serum hs-CRP levels than LVH- patients (both P < 0.05, Table 1). However, the serum apelin level was dramatically lower in LVH+ patients (P < 0.001). Pears correlation analyses show a slight correlation between serum apelin and age and that there is no correlation between serum apelin and sex, BNP and hs-CRP (data not shown).

**Table 1:** The clinical and biochemical characteristic of all enrolled patients

Variables	LVH+ (139)	LVH− (288)	P value
Age (years)	54.4 ± 6.3	54.5 ± 9.1	0.861
Male (n, %)	85 (61.2 %)	171 (59.4 %)	0.404
Smoker (n, %)	42 (42.9 %)	70 (31.7 %)	0.001
BMI (kg/m^2^)	24.7 ± 2.5	24.3 ± 1.6	0.036
SBP(mmHg)	161.1 ± 12.4	152.2 ± 9.3	<0.001
DBP (mmHg)	84.2 ± 5.8	85.1 ± 5.7	0.111
TG (mmol/L)	1.7 ± 0.7	1.8 ± 0.6	0.069
TC (mmol/L)	5.0 ± 0.9	5.1 ± 0.9	0.394
HDL-C (mmol/L)	1.9 ± 0.7	1.8 ± 0.6	0.161
LDL-C(mmol/L)	3.0 ± 0.9	2.9 ± 1.0	0.401
sCr (mmol/L)	107.1 ± 31.3	105.3 ± 31.2	0.677
hs-CRP(mmol/L)	2.3 ± 0.8	1.7 ± 0.7	<0.001
Serum apelin (ng/mL)	1.7 ± .0.7	2.4 ± 0.7	<0.001

*BMI* body mass index, *SBP* systolic blood pressure, *DBP* diastolic blood pressure, *TG* total triglyceride, *TC* total cholesterol, *HDL* high-density lipoprotein cholesterol, *LDL* low-density lipoprotein cholesterol, *SCR* serum creatinine, *Hs-CRP* high sensitive-C-reactive protein

**Fig. 1:**
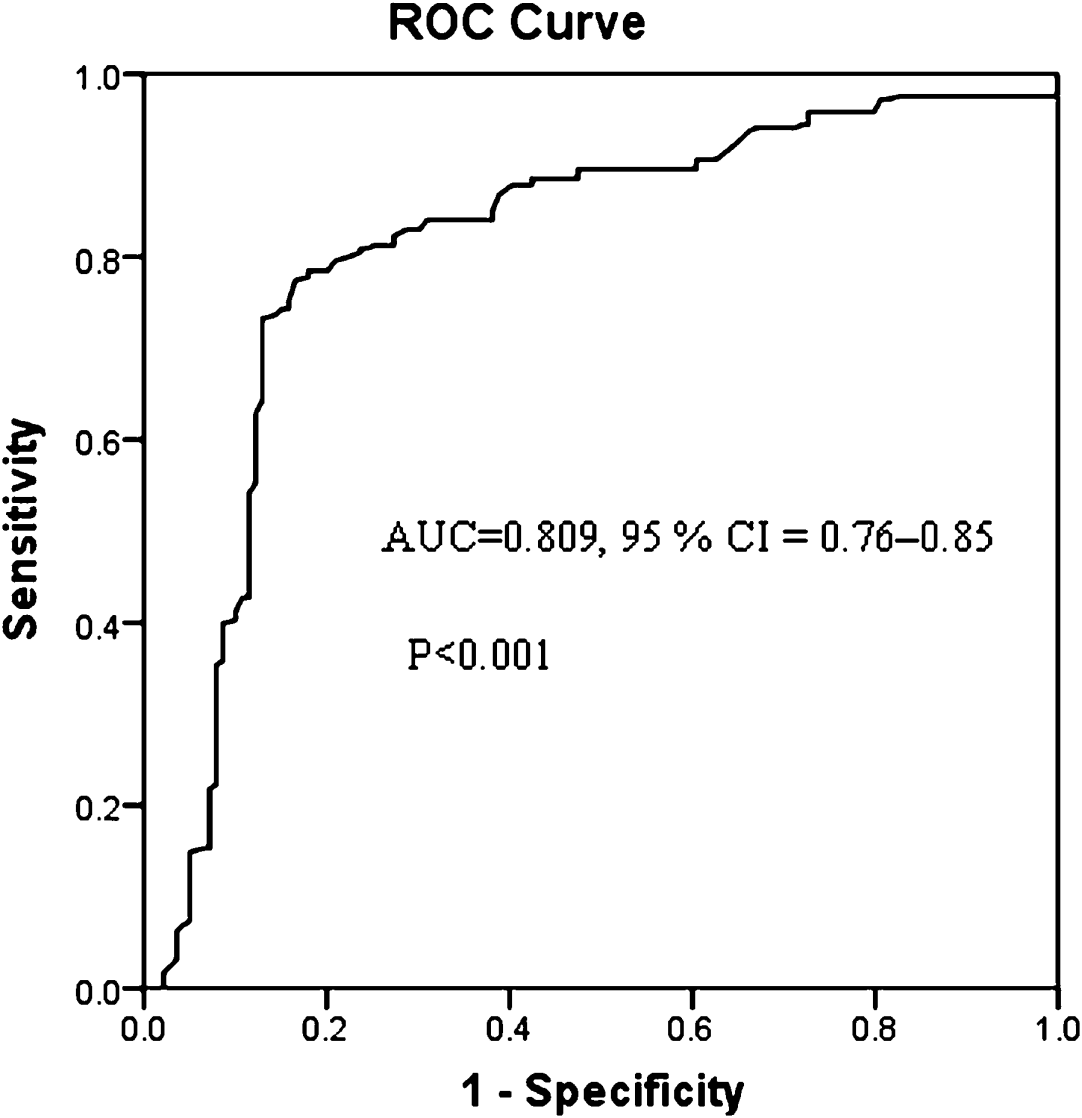
The ROC curve of serum apelin level. Serum apelin at 1.92 ng/mL is excellent to discriminate LVH prevalence in hypertensive patients, with 78 specificity and 82 % sensitivity. The AUC value was 0.809 (95 % CI = 0.76 to −0.85, P < 0.001)

Using ROC curve and area under curve [15], we determined the sensitivity and specificity of apelin as a diagnostic marker of the prevalence of LVH in hypertensive patients. A ROC curve was created to estimate the serum APLN level with the highest sensitivity and specificity. Figure 1 shows that serum apelin is able to discriminate LVH+ patients from the LVH− patients. Our ROC analysis data revealed that serum apelin levels were robust in discriminating patients with LVH from those without, with an AUC value of 0.809 (95 % CI = 0.76–0.85, P < 0.001). The overall best cut-off value for apelin protein was estimated as 1.92 ng/mL with 78 % specificity and 82 % sensitivity.

The multivariate logistic regression using LVH+/LVH− as the dependent variable and serum apelin as a covariate adjusted for conventional risk factors listed in Table 1 show that the serum apelin is a protective factor against LVH prevalence (OR = 0.16, 95 % CI: 0.11–0.25, P < 0.001). Another factor associated with LVH was serum hs-CRP level (OR = 3.1, 95 % CI: 2.1–4.5, P < 0.001). Other factor, such as age, gender, BMI, diabetes, smoking, SBP, and serum lipids (HDL- and LDL-cholesterol, were not associated with LVH prevalence.

**Fig. 2:**
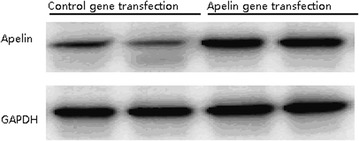
The apelin protein detection in cultured cardiomyocytes after apelin gene and control transfection by Western blot assay. Our results showed that the gene transfection caused a markedly increase in the apelin level compared to control cells

We next detected apelin protein expression in cultured cardiomyocytes receiving apelin gene and control transfection. Western blot assay showed that the apelin gene transfection dramatically increased the apelin level compared to control cells (Fig. 2).

**Fig. 3:**
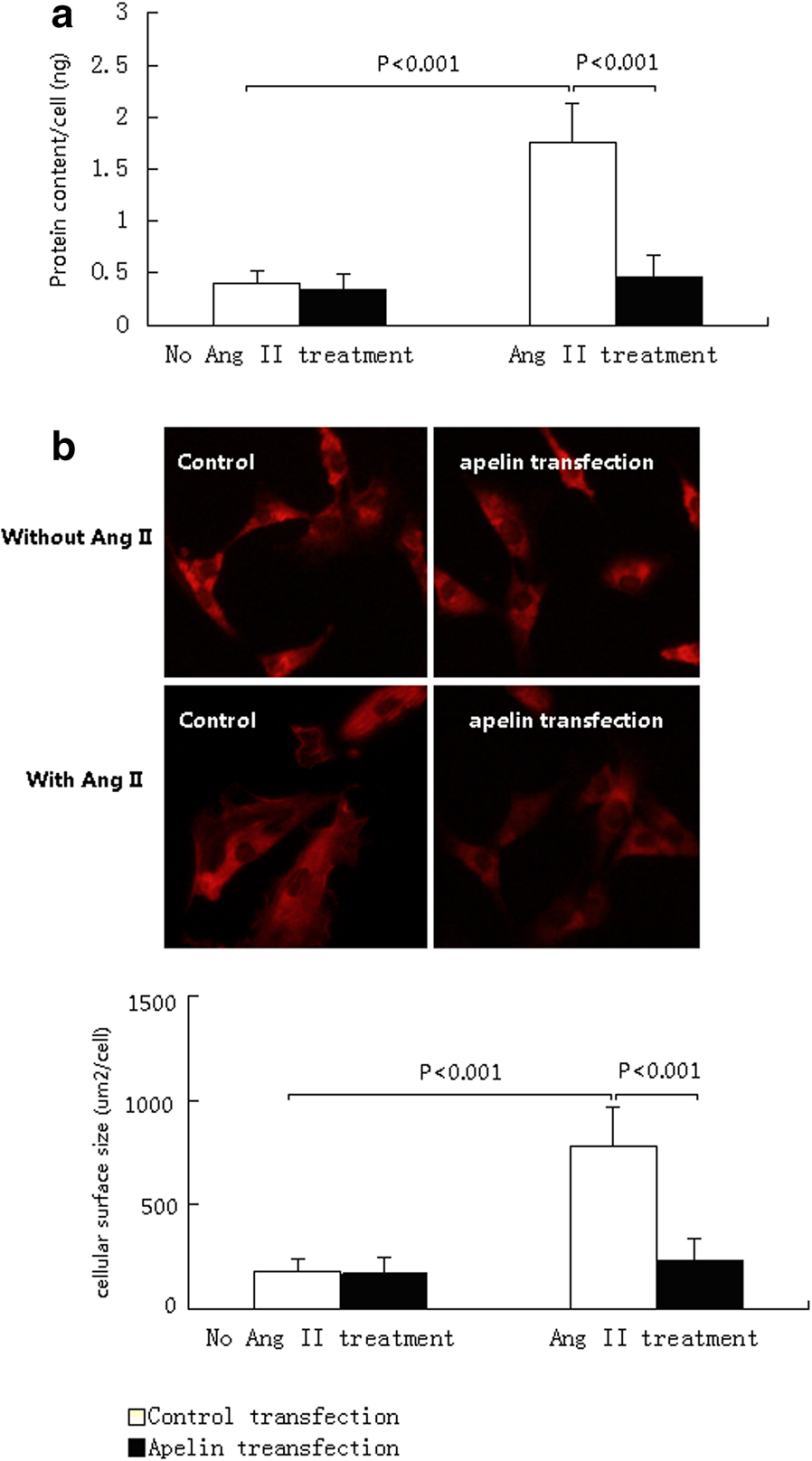
The hypertrophic changes of cultured cardiomyocytes over-expression apelin and controls. The hypertrophic changes of cells were indicated by the cellular protein content and cellular surfaces sizes. **a** Cultured cardiomyocytes had similar cellular protein contents between control and apelin gene transfection cells. However, when treated with Ang II, the cells over-expressing apelin had significantly lower protein contents. **b** The typical images of cellular size before and after Ang II treatment in cultured cardiomyocytes over-expression apelin and controls. Cardiomyocytes had similar cellular sizes between control and apelin gene transfected cells. After Ang II treatment, the cells over-expressing apelin had significantly smaller size than controls

Our in vitro study showed that the cellular protein content and surfaces were similar between cardiomyocyte over-expressing apelin and control cells in the absence of Ang II treatment. However, when cells were treated with Ang II, control cells without apelin over-expression had significantly increased in cellular protein content and surfaces. In contrast, the cellular protein content and surfaces in apelin over-expressing cardiomyocytes did not increase markedly (Fig. 3a, b).

**Fig. 4:**
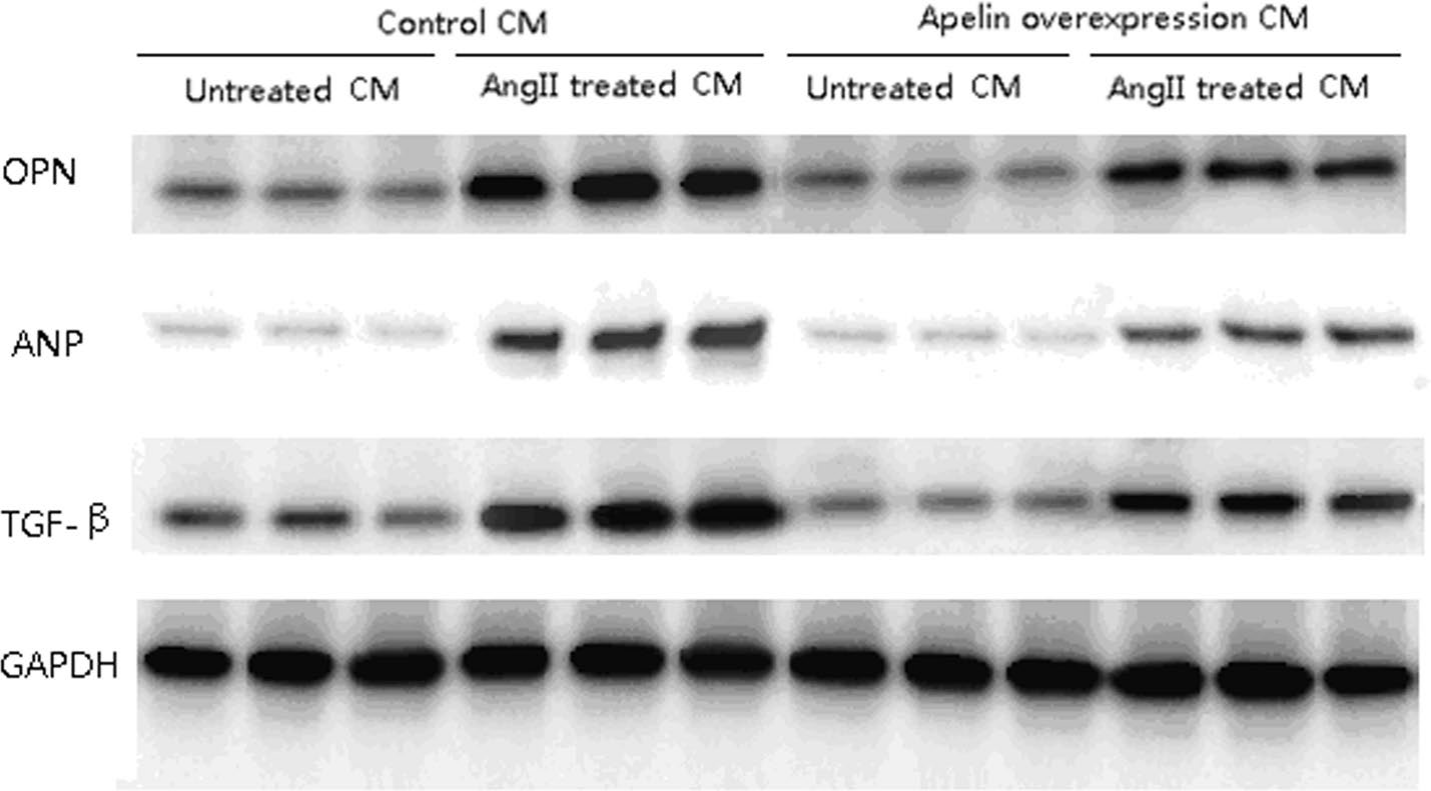
The expressions of several cardiac hypertrophy markers in cardiomyocytes. Ang II induced dramatically increases of Osteopontin (OPN), atrial natriuretic peptide (ANP), and transforming growth factor-β (TGF-β), which were inhibited by apelin over-expression

Figure 4 shows Ang II treatment induced dramatically increases of the several cardiac hypertrophy markers, such as OPN, ANP, and TGF-β in cardiomyocytes. As we expected, we observed that the apelin over-expression abolished the Ang II induced increases in these hypertrophy marker expressions in cardiomyocytes (Fig. 4).

## Discussion

The present study demonstrated significantly lower serum apelin level in hypertensive patients with echocardiographic evidence of LVH compared with hypertensive patients without LVH. It also showed an independent association of low apelin level with LVH prevalence after adjusting for other confounding clinical parameters.

LVH is considered to be a critical intermediate phenotype in the progression of hypotensive heart disease and is related to adverse outcomes [16]. Pathological changes in patients with hypertensive LVH consist of increased size of the cardiomyocyte, and altered extracellular matrix with accumulation of fibrosis [17]. There is considerable interindividual variability observed in the development and pattern of LVH [18, 19]. While extensive efforts have been made to identify putative genetic risk factors that could modulate LVH, including candidate gene association studies and genome-wide association studies [20, 21], these have yet to yield clinically applicable results.

The involvement of the apelin–APJ system in hypertension has been studied extensively in the past few years [22, 23], nevertheless, in the setting of hypertensive patients with LVH, there is little knowledge about the role of apelin. Przewlocka-Kosmala et al. have documented lower apelin levels in hypertensive patients with more severe left ventricular systolic and diastolic function abnormalities than their peers with higher apelin levels [24]. Notably, those patients had used antihypertensive drugs before the study; therefore, the influence of pharmacotherapy on the results cannot be entirely ruled out.

In line with the observation mentioned above, the result presented here showed that low serum apelin levels were associated with LVH prevalence in untreated hypertensive patients. Additionally, the association between serum apelin and LVH prevalence in several multivariate models remained statistically significant after adjustments for the major commonly recognized risk factors, including BMI, systolic blood pressure and serum hs-CRP. Furthermore, we used neonatal rat cardiomyocytes to directly test the effects of apelin on cardiac hypertrophy induced by AngII. In accordance with our clinical data, in vitro results demonstrated that overexpression of apelin could attenuate the AngII-mediated increase in cell size, protein content and the expression of pro-hypertrophic and/or pro-fibrotic factors, including TGF-b, OPN and ANP [25–27]. These findings are also consistent with previous findings showing that Apelin-APJ signaling could counteract the effects of AngII. For instance, administration of apelin blocks a spectrum of AngII-mediated effects on atherosclerosis in the apolipoprotein E-deficient mice through the formation of a heterodimer between the apelin and AngII receptors [7]. Apelin also protects wild type mice against AngII-induced hypertension and cardiovascular fibrosis via direct regulation of PAI-1 gene expression [28].

A substantial body of evidence supports the concept that apelin could prevent cardiovascular remodeling induced by myocardial infarction or by pressure overload in mice or rats through different mechanisms [12, 25, 29–33]. In addition, it has previously been suggested that apelin causes vasodilation in animal models as well as in clinical studies [34, 35]. Moreover, experimental evidence indicates that apelin is among the most potent endogenous inotropic agents [22]. Apelin induces angiogenesis in post-myocardial infarction of diabetes [36]. Apelin gene therapy increases myocardial vascular density and ameliorates diabetic cardiomyopathy via upregulation of sirtuin [5].

Several limitations should be addressed in this study. Firstly, a larger scale study with serial assessment of ventricular structure and function would be desirable. Secondly, this study was a cross-sectional study, not a longitudinal study. A follow-up is needed to evaluate the relation between apelin and LVH incidence and regression. Thirdly, it would be interesting for future studies to demonstrate the association between all forms of apelin and LVH.

## Conclusion

In conclusion, we have reported that serum apelin level is strongly and independently related to LVH in a population with essential hypertension, suggesting that this peptide may be used as a biomarker of LVH. In addition, considering apelin’s vasodilating and inotropic properties, the apelin system may lead to the development of new therapeutic regimens, both for lowering blood pressure and improving myocardial performance.
